# Relationship between left ventricular diastolic function and geometric patterns in Nigerians with newly diagnosed systemic hypertension

**Published:** 2009-05

**Authors:** Umar G Adamu, Philip M Kolo, Ibrahim A Katibi, George O Opadijo, Ayodele BO Omotosho, Matthew A Araoye

**Affiliations:** Department of Medicine, University of Ilorin Teaching Hospital, Ilorin, Nigeria; Department of Medicine, University of Ilorin Teaching Hospital, Ilorin, Nigeria; Department of Medicine, University of Ilorin Teaching Hospital, Ilorin, Nigeria; Department of Medicine, University of Ilorin Teaching Hospital, Ilorin, Nigeria; Department of Medicine, University of Ilorin Teaching Hospital, Ilorin, Nigeria; Department of Medicine, University of Ilorin Teaching Hospital, Ilorin, Nigeria

## Abstract

**Background:**

It is not known whether abnormalities of left ventricular diastolic function are influenced by the various cardiac geometric patterns in Nigerians with newly diagnosed systemic hypertension.

**Objective:**

To evaluate the relationship between the parameters of left ventricular diastolic function and the geometric patterns in this group of patients.

**Methods:**

Two-dimensional, guided M-mode echocardiography including Doppler was performed in 150 consecutive, newly diagnosed hypertensive individuals and normotensive controls aged between 35 and 74 years. Left ventricular mass index and relative wall thickness were used to classify the hypertensive individuals into four geometric patterns, and the pulsed-wave Doppler parameters obtained were used to categorise the abnormalities of diastolic function.

**Results:**

Four left ventricular geometric patterns were identified: 23 (15.3%) had normal left ventricle geometry, 33 (22%) had concentric remodelling, 37 (24.7%) were found to have eccentric hypertrophy, and concentric hypertrophy occurred in 57 (38%) of the hypertensive individuals. Left ventricular diastolic dysfunction occurred more in hypertensives with concentric left ventricular geometric pattern. Increased left ventricular mass index and relative wall thickness were found to be associated with the mitral E-wave, E/A ratio and pulmonary venous flow S-wave in the hypertensives (*p* < 0.001).

**Conclusion:**

In newly diagnosed Nigerian hypertensives, the abnormalities in left ventricular diastolic function varied between the different left ventricular geometric patterns, being worst in those with concentric geometry.

## Summary

Left ventricular hypertrophy (LVH) is a known complication of systemic hypertension (SH) and has long been recognised as an independent risk factor for adverse outcomes in individuals with systemic hypertension.^1^ The adaptation of the left ventricle (LV) to uncontrolled SH takes various forms, which can be described in four different geometric patterns using the combinations of left ventricular mass index (LVMI) and relative wall thickness (RWT). They are: normal geometry with normal LVMI and RWT; concentric remodelling, normal LVMI and increased RWT; eccentric hypertrophy, increased LVMI and normal RWT; and concentric hypertrophy, with increased LVMI index and RWT.^2^ Many studies have shown that the incidence of cardiovascular events is greatest in patients with concentric LVH, intermediate in those with eccentric and concentric remodelling geometric patterns, and least in patients with normal geometry.

Left ventricular diastolic function has been well documented in Caucasians with SH complicated with LVH.^3^-^5^ Many authors assume that the changes in hypertensive blacks with LVH may be different from what one sees in Caucasians. However, no information is currently available to justify such a claim or to demonstrate the relationship between LV diastolic function and LV geometric patterns in Nigerians with SH. Consequently, this study was designed to address that issue.

## Methods

The study involved 150 consecutive, newly diagnosed adult Nigerians with SH aged between 35 and 74 years (mean age 52.9 ± 9.8 years, 76 women). They were seen at the cardiology clinic of the University of Ilorin Teaching Hospital. Their blood pressure (BP) was measured with a mercury (Accoson’s) sphygmomanometer in the sitting position in the consulting room after 10 minutes of rest, according to standard guidelines.^6^ Systolic and diastolic BP were taken at Korotkoffs sounds I and V, respectively. A patient was considered to have SH and was enrolled in the study if his/her systolic and diastolic BP averaged ≥ 140 and ≥ 90 mmHg, respectively on three hospital visits at one-week intervals. Other clinical information obtained included the weight and height, which were used to calculate the body mass index, as well as waist and hip circumference and the waist-to-hip ratio. All parameters were obtained before commencing treatment.

One hundred and fifty age- and gender-matched healthy normotensive (BP < 140/90 mmHg) controls were recruited from individuals on routine medical check-up, patients’ relatives and the hospital staff. Excluded from this group were individuals with a history of diabetes mellitus, congestive cardiac failure, chronic renal failure, coronary artery disease, thyrotoxicosis, moderate to severe obesity and chronic alcohol abuse. Pregnant women and patients with atrial fibrillation were also excluded. The study protocol was approved by the ethical review committee and written informed consent was obtained from all those who participated in the study.

## Echocardiography

All the 300 subjects (150 patients with SH and 150 normal controls) underwent transthoracic two-dimensional guided Mmode and Doppler echocardiography. The echocardiograms were obtained with a commercially available cardiac ultrasound machine (ESAOTE SPA Megas CVS, Italy) equipped with a 2.5-MHz transducer in the parasternal and apical views with the subjects in the left lateral decubitus position. LV measurements were obtained at end-diastole and end-systole, according to the American society of echocardiography (ASE) recommendations.^7^ The measurements taken included the interventricular septal thickness in systole (IVSs) and in diastole (IVSd), posterior wall thickness in systole (PWs) and in diastole (PWd), and LV internal dimension in systole (LVDs) and in diastole (LVDd). The measurements were the average of three cardiac cycles.

## Calculation of derived variables

LV mass was achieved using the modified Devereux formula:^8^

LV mass (ASE) = 0.8 [1.04 (IVSd + PWd + LVDd)^3^ – LVDd^3^] + 0.6.

LV mass was indexed to body surface area to obtain LVMI.^9^ LVH was considered present when LVMI was more than 134 g/m^2^ in men and more than 110 g/m^2^ in women.^10^ The relative wall thickness was calculated as twice the posterior wall thickness/LV end-diastolic diameter, and increased RWT was when the ratio was above 0.44.^1^,^8^

Accordingly, the combinations of LVMI and RWT values were used to define four LV geometric patterns as follows; normal geometry, composed of both normal LVMI and RWT; concentric remodelling: increased RWT but normal LVMI; eccentric hypertrophy: increased LVMI and normal RWT; and the increase in both variables in identified hypertensive individuals with concentric LVH.^2^,^11^

## Doppler indices of diastolic function

LV diastolic function was evaluated by pulsed-wave Doppler recordings of both the transmitral inflow and pulmonary venous flow velocities in the apical four-chamber view.^12^ The transmitral velocities were recorded by placing the cursor between the tips of the opened mitral valve leaflets during maximal opening, as nearly parallel to the flow as possible, to capture the maximal velocity during diastole. The maximal velocities of three consecutive cardiac cycles were traced manually and averaged.

The variables measured include the early filling (E-wave) and late (A-wave) diastolic peak filling velocities. From these, the E-wave to A-wave (E/A ratio) velocity was calculated. The Valsalva manoeuvre was performed where applicable. The mitral deceleration time (DT) was measured as the time interval from the peak to the end of the E-wave. Isovolumic relaxation time (IVRT) was measured as the interval between the aortic valve closure click to the beginning of the transmitral inflow with the simultaneous visualisation of the aortic and mitral flows.^13^

The pulmonary venous velocity was recorded by placing the cursor 1 to 2 cm into the right upper pulmonary vein, close to the interatrial septum. In difficult cases this was achieved with the guidance of colour Doppler. The peak systolic (S-wave) and diastolic (D-wave) forward velocities and the peak atrial reversal (Ar) wave velocity were recorded, and the ratio of S/D was calculated. Where the pulmonary S-wave was biphasic, the higher peak velocity was used. LV diastolic dysfunction was categorised according to its clinical severity into normal, impaired relaxation, pseudonormal and the restrictive pattern.^14^

## Statistical analysis

Data analysis was performed with SPSS software (SPSS, Inc, Chicago, Illinois, USA). Continuous variables were expressed as mean ± SD and categorical variables as percentages. Comparison between the means of independent samples was performed with the Student’s *t*-test and comparisons between the different LV geometric patterns were done with analysis of variance (ANOVA). Scheffé’s test was used for *post hoc* analyses. The Pearson correlation was used to evaluate the relationship between two parameters. Linear regression analysis using an enter method was applied to determine the effects of cardiac parameters (LVMI, RWT and PWd) as determinants of parameters of LV diastolic function. A two-tailed value of *p* < 0.05 was considered significant.

## Results

A total of 300 subjects were involved in the study; 150 were patients with SH and 150 were age- and gender-matched normotensive controls. They were aged between 35 and 74 years, with a mean age of 52.74 ± 9.8 for the patients, compared to 52.59 ± 9.6 for the control group (*p* > 0.896) (Table 1). The mean systolic and diastolic BP was significantly higher in the hypertensive individuals than in the normotensive controls (*p* < 0.001). The two groups were, however, comparable in the mean values for age, body mass index and heart rate. The demographic and echocardiographic characteristics of the different geometric patterns in patients are shown in Table 2.

**Table 1:** Demographic And Clinical Characteristics Of The Study Population

	*Hypertensives (n = 150)*	*Normotensives (n = 150)*	p*-value*
Age (years)	52.74 ± 9.81	52.59 ± 9.58	0.896
Gender (male/female)	74 /76	74/76	0.172/0.233
BMI (kg/m^2^)	27.82 ± 4.23	27.25 ± 3.10	0.228
WHR	0.91 ± 0.05	0.90 ± 0.05	0.400
SBP (mmHg)	162.00 ± 20.88	115.51 ± 10.61	< 0.001*
DBP (mmHg)	98.33 ± 13.12	73.80 ± 7.82	< 0.001*
HR (bpm)	74.3 ± 6.3	73.5 ± 9.3	0.396
Pulse pressure (mmHg)	63.37 ± 19.59	41.37 ± 11.01	< 0.001*

BMI: body mass index, DBP: diastolic blood pressure, SBP: systolic blood pressure, HR: heart rate, bpm: beats per minute, WHR: waist-hip ratio, plus-minus values are means ± SD, *statistically significant

**Table 2:** Demographic And Echocardiographic Characteristics Of The Different LV Geometric Patterns

*Variables*	*NC (n = 150)*	*NG (n = 23)*	*CR (n = 33)*	*EG (n = 37)*	*CG (n = 57)*	*Overall p-value*	p*-value between NC and NG*	p*-value in hypertensives*
Age (years)	52.6 ± 9.6	52.7 ± 10.1	52.52 ± 10.2	53.3 ± 10.5	52.3 ± 9.2	0.997	0.998	0.980
BMI (kg/m^2^)	27.3 ± 4.0	25.7 ± 4.2	28.1 ± 4.5	28.5 ± 4.4	28.1 ± 3.8	0.06	0.085	0.006*
SBP (mmHg)	115.5 ± 10.6	158.4 ± 13.7	155.2 ± 15.5	163.8 ± 17.1	166.3 ± 26.6	0.001	0.001*	0.076
DBP (mmHg)	73.8 ± 7.8	94.9 ± 13.7	95.9 ± 9.5	99.6 ± 12.2	100.3 ± 15.0	0.001	0.001*	0.229
Pulse pressure (mmHg)	41.4 ± 11.0	65.2 ± 17.7	58.2 ± 17.7	64.3 ± 20.6	65.1 ± 20.7	0.001	0.001*	0.394
Heart rate (bpm)	73.3 ± 9.3	73.7 ± 5.5	73.7 ± 6.8	73.5 ± 6.8	74.3 ± 5.9	0.824	0.592	0.930
LVDd (cm)	4.6 ± 5.3	4.8 ± 0.6	4.2 ± 0.6	5.1 ± 0.5	4.5 ± 0.5	0.001	0.143	0.001*
LVDs (cm)	3.1 ± 0.5	3.2 ± 0.5	2.9 ± 0.6	3.4 ± 0.6	2.9 ± 0.5	0.001	0.565	0.001*
PWd (cm)	0.93 ± 0.15	0.83 ± 0.14	1.08 ± 0.16	0.95 ± 0.18	1.31 ± 0.24	0.001	0.002*	0.001*
IVSd (cm)	1.01 ± 0.20	1.11 ± 0.35	1.22 ± 0.28	1.47 ± 0.30	1.49 ± 0.30	0.001	0.056	0.001*
RWT	0.41 ± 0.09	0.35 ± 0.08	0.57 ± 0.09	0.36 ± 0.06	0.61 ± 0.12	0.001	0.002*	0.001*
EF (%)	64.2 ± 9.3	61.6 ± 8.8	61.8 ± 4.9	62.0 ± 8.8	63.6 ± 7.4	0.365	0.202	0.623
FS (%)	33.3 ± 5.5	33.7 ± 7.1	32.6 ± 7.5	34.4 ± 7.5	34.8 ± 7.3	0.445	0.181	0.543
LA (cm)	3.19 ± 0.42	3.45 ± 0.76	3.21 ± 0.45	3.58 ± 0.23	3.48 ± 0.58	0.001	0.016*	0.001*
LVMI (g/m^2^)	97.3 ± 16.5	102.9 ± 19.5	105.9 ± 15.5	157.8 ± 40.6	165.6 ± 44.2	0.001	0.134	0.001*

IVSd: interventricular septal thickness in diastole, LVDd: left ventricular dimension in diastole, LVDs: left ventricular dimension in systole, PWd: posterior wall thickness in diastole, EF: ejection fraction, FS: fractional shortening, LAD: left atrial dmension, ARD: aortic root dimension, RST: relative septal thickness, RWT: relative wall thickness, LVMI: left ventricular mass index, plus-minus values are means ± SD, NC: normal control, NG: normal geometry, CR: concentric remodelling, eccentric geometry, CG: concentric geometry, * statistically significant.

LVMI was increased in the patient subgroups with concentric and eccentric geometry compared to the controls (*p* < 0.001). It varied within the subgroups, being higher in patients with concentric geometry than in those with eccentric geometry. The RWT also increased significantly (*p* < 0.001) in subjects with concentric geometry and concentric remodelling compared with the controls. Patients with the four geometric patterns and the normotensive controls were similar regarding the fractional shortening (*p* = 0.445) and ejection fraction (*p* = 0.365).

## Parameters of LV diastolic function

Table 3 shows the mean values of the parameters of LV diastolic function in the study group. Satisfactory recordings were achieved in 88% of newly diagnosed cases of SH and in 93% of the controls. LV diastolic function was found in 62% of the hypertensive subgroups. About 38% of the patients showed normal diastolic function, while 52.7% had impaired relaxation. The pseudonormal pattern was found in 8%, whereas the remaining 1.3% had the restrictive pattern. The mean values for the Ewave, E/A ratio and IVRT in patients with the different geometric patterns differed significantly when compared with the controls (*p* < 0.001). The mean S-wave (*p* < 0.009) and S/D ratio (*p* < 0.003) also differed significantly between the study groups. No statistically significant difference was observed between the subgroups with regard to the mean A-wave (*p* = 0.852) and the mean Ar wave (*p* = 0.483). In a subgroup analysis, the E-wave, E/A ratio and IVRT differed significantly between those with concentric geometry and the controls (*p* < 0.001).

**Table 3:** Left Ventricular Diastolic Function Parameters In The Different Geometric Patterns

*Variables*	*NC (n = 150)*	*NG (n = 23)*	*CR (n = 33)*	*EG (n = 37)*	*CG (n = 57)*	*Overall p-value*	p*-value between NC and NG*	p*-value in hypertensives*
E-wave (ms)	68.4 ± 15.6	54.0 ± 14.0	59.2 ± 12.3	59.4 ± 13.12	56.9 ± 14.6	0.001*	0.001*	0.415
A-wave (ms)	56.2 ± 43.4	59.9 ± 19.6	61.5 ± 17.2	61.6 ± 16.3	59.1 ± 14.7	0.852	0.692	0.871
E/A	1.33 ± 0.27	0.96 ± 0.26	1.03 ± 0.32	1.03 ± 0.31	1.02 ± 0.41	0.001*	0.001*	0.856
IVRT (ms)	83.8 ± 16.0	95.0 ± 26.1	96.2 ± 26.6	94.4 ± 26.0	98.0 ± 30.5	0.001*	0.004*	0.937
DT (ms)	177.5 ± 40.7	156.7 ± 57.2	168.9 ± 58.5	152.8 ± 58.4	159.8 ± 30.5	0.027*	0.034*	0.731
S-wave (ms)	40.6 ± 11.2	32.4 ± 9.7	36.8 ± 10.9	35.9 ± 9.9	34.0 ± 10.8	0.009*	0.011*	0.533
D-wave (ms)	33.7 ± 10.3	27.5 ± 7.3	32.8 ± 9.3	35.4 ± 10.1	31.4 ± 8.8	0.096	0.030*	0.060
Atrial reversal (ms)	22.4 ± 5.1	20.6 ± 5.6	23.6 ± 6.1	23.0 ± 3.9	22.5 ± 5.5	0.483	0.228	0.360
S/D	1.26 ± 0.35	1.22 ± 0.34	1.15 ± 0.28	0.98 ± 0.28	1.1 ± 0.27	0.003*	0.654	0.055

IVRT: isovolumic relaxation time, Ar: atrial reversal, S: systolic wave, D: diastolic wave, S/D: systolic and diastolic wave ratio, plus-minus values are means ± SD, NC: normal control, NG: normal geometry, CR: concentric remodelling, EG: eccentric geometry, CG: concentric geometry, *statistically significant.

## Left ventricular geometry and diastolic dysfunction pattern

In the patients with SH, 85% showed an altered geometry pattern: 22% with concentric remodelling, 25% with eccentric hypertrophy and 38% with concentric hypertrophy. None of the controls showed severe forms of altered geometry or abnormal LV diastolic function. The patients with concentric LVH had the highest incidence of diastolic dysfunction, with 56.1% of them having impaired relaxation, the pseudonomal pattern occurred in 3.5%, and only 1.8% had the restrictive pattern. None of the patients with a normal geometry pattern demonstrated the restrictive pattern.

## Correlation

Correlation coefficients were calculated between some of the LV diastolic function parameters as dependent variables and LVMI, RWT and posterior wall thickness as independent variables after accounting for the confounding factors age, gender, blood pressure and heart rate (Table 4). In univariate analysis, the E-wave and E/A ratio correlated significantly but inversely with posterior wall thickness (*p* < 0.001), LVMI (*p* < 0.001) and relative wall thickness (*p* < 0.01). There was no significant correlation between the A-wave and the above independent variables (*p* > 0.05). Also, IVRT did not relate to LVMI (*p* = 0.331), PWd (*p* = 0.639) and RWT (*p* = 0.547). The S-wave and the S/D ratio correlated negatively with LVMI (*p* < 0.001 and *p* < 0.033, respectively). Multiple regression analysis (Table 5) yielded R^2^ = 0.134; *p* < 0.001 between the E-wave, pulmonary venous S-wave and E/A ratio and the LVMI and RWT.

**Table 4:** Univariate Correlates Of Posterior Wall Thickness In Diastole, RWT, LVMI

	*PWd*		*RWT*		*LVMI*	
*Variables*	*R*	p*-value*	*R*	p*-value*	*R*	p*-value*
E-wave (ms)	–0.2073	< 0.01*	–0.2038	< 0.01*	–0.3017	< 0.001*
A-wave (ms)	–0.1383	0.086	0.1022	0.200	0.0980	0.225
E/A ratio	–0.2694	< 0.001*	–0.1893	0.018*	–0.3104	< 0.001*
Isovolumic relaxation rate	0.0380	0.639	0.0487	0.547	0.0786	0.331
E-wave deceleration time	0.0506	0.532	0.0539	0.433	–0.0026	0.975
Pulmonary S-wave	–0.1736	0.031*	–0.1219	0.131	–0.2300	< 0.001*
Pulmonary D-wave	–0.0857	0.289	–0.0756	0.350	–0.0883	0.274
Atrial reversal	0.0377	0.642	0.0473	0.559	–0.0163	0.840
Pulmonary S/D ratio	–0.1001	0.215	–0.0441	0.585	–0.1718	0.033

E-wave: mitral early filling wave, A-wave: mitral atrial filling wave, pulmonary S-wave: pulmonary systolic wave, pulmonary D-wave: pulmonary diastolic wave, PWd: posterior wall thickness, RWT: relative wall thickness, LVMI: left ventricular mass index, *statistically significant.

**Table 5:** Multiple Linear Regression Analysis Of LVMI As Dependent Variable And E-Wave And E/A Ratio As Independent Variables

LVMI	*R* = 0.367	*R*^2^ = 0.134	Adjusted	*R*^2^ change = 0.134	*p* < 0.001*
Coefficients	β	SE	standard β	*t*	
E-wave	–0.615	0.162	–0.236	–0.3785	*p* < 0.001*
E/A ratio	–38.308	7.091	–0.328	–0.5402	*p* < 0.001*
Pulmonary S-wave	–0.908	0.317	–0.225	–2.865	*p* < 0.001*

E-wave: mitral early filling wave, A-wave: mitral atrial filling wave, pulmonary S-wave: pulmonary systolic wave, LVMI: left ventricular mass index, *statistically significant.

## Discussion

The purpose of this study was to evaluate LV diastolic function with a view to highlighting its association with the different geometric patterns in Nigerians with newly diagnosed SH. Our results showed that the LV diastolic function, as reflected by the combinations of transmitral and pulmonary venous flow velocities, was common in newly diagnosed and untreated SH patients compared with normotensive controls. Secondly, concentric hypertrophy was the commonest pattern of LVH and also demonstrated the greatest proportion of LV diastolic dysfunction. Thirdly, the LVDF abnormalities were related to cardiac structural changes.

The combined use of LVMI and RWT enabled the distinction of four geometric patterns. Ganau *et al*.^2^ divided the LV adaptive changes in Caucasians into four geometric patterns, including a normal pattern. In their study, concentric hypertrophy occurred in 8% of patients while eccentric LVH was observed in 27% of the middle-aged hypertensives with LVH. By contrast, in Nigerians, Aje *et al*.^15^ observed that 28% of the newly presenting SH patients exhibited concentric LVH while 18% demonstrated the eccentric geometric pattern. This study corroborates the previous finding that concentric LV geometry was the commonest pattern, having occurred in 38% of our study population. In contrast, eccentric LVH was the predominant geometric pattern seen in the study by Ganau *et al*.^2^

It has been suggested that the increase in LVMI in Caucasians is related to the presence of coronary artery disease and tends to compensate for the loss of contractile elements. In SH patients, concentric LVH predominates to compensate for the pressure overload, thereby preserving systolic function. In addition to environmental and genetic factors, it appears that eccentric LVH, being a powerful coronary artery disease risk factor, may have been present even before the ischaemic event.^16^ In this study, patients with coronary artery disease were not included because the disease is rare in this area. It could also be assumed, therefore, that the alterations observed in our study resulted from untreated SH.

Previous studies in Caucasians have reported the relationship between LV geometric patterns and LV diastolic function.^1^,^3^-^5^ In a study involving mild-to-moderate SH, LV diastolic function was impaired significantly in patients both with concentric and eccentric LVH.^3^ In the LIFE study by Wachtell *et al*.,^4^ LV diastolic function differed between the different geometric patterns in patients, with concentric LVH demonstrating the most severe impairment. In that study, IVRT and A velocity were higher in patients with concentric geometry.

In an echocardiographic study of a large sample of patients with SH, Cloffi *et al.* found the E/A ratio to be lower in patients with concentric LV geometry. This was attributed to a higher late transmitral gradient, the hallmark of greater left atrial ejection force.^17^ Our findings of significantly lower E/A ratios and Ar in patients with concentric LV geometry lends support to that of Cloffi *et al.* 17 It suggests that the pressure overload in SH patients significantly affects relaxation and is greater in the presence of concentric LV geometry. The fibrosis and subsequent extracellular matrix disarray and the microcirculation might also affect normal LV relaxation.

## Limitations of the study

The study enrolled only hypertensive individuals with mild obesity to minimise the contribution of more severe forms of obesity to LVH. This would have eliminated hypertensive individuals with greater LVM and consequent LVDD. Although CAD is not very common in patients in our environment and was excluded using only clinical examination and electrocardiography, the use of treadmill exercise electrocardiography and coronary angiography would have helped to conclusively exclude CAD. It is also possible that the so-called newly diagnosed hypertensive individuals might have been previously diagnosed but were not compliant with taking their medication.

## Conclusion

This study has shown that LV diastolic dysfunction, which is an important cause of morbidity in SH patients, differed between the four LV geometric patterns. Concentric LVH was the predominant pattern in Nigerians with SH. Also, LVMI and RWT were associated with low E/A ratios and the Ar velocity of pulmonary venous flow.
